# Supplementary material for: TRPM7-Mediated Ca2+ Regulates Mussel Settlement through the CaMKKβ-AMPK-SGF1 Pathway
Source: Int J Mol Sci. 2023 Mar 11;24(6):5399. doi: 10.3390/ijms24065399 (PMC10049526; doi:10.3390/ijms24065399)
Supplement: Supplementary file 1 [file ijms-24-05399-s001.zip › ijms-2189154-supplementary.pdf]

Supplementary Materials

Methods

Construction of cDNA Expression Library

Total RNA from pediveliger larvae of *Mytilopsis sallei* was extracted with a TRIzol RNA Kit (Invitrogen) following the manufacturer's instructions, and 70 μg of total RNA was used to isolate mRNA. The integrity of the total RNA was detected by separating RNA species on a 1% agarose gel, and the quantity was determined with a NanoDrop ND-2000 UV-Visible Spectrophotometer (Thermo Fisher Scientific). The mRNA isolated with an Oligotex mRNA Midi Kit (QIAGEN, Hilden, Germany) was used for cDNA library construction.

A CloneMiner II cDNA Library Construction Kit (Thermo Fisher Scientific) was used to construct the cDNA library. Briefly, reverse transcription using the primer Biotin-*att*B2-Oligo(dT), synthesis of double-stranded cDNA, fractionation followed by connection with an *att*B1-adapter, base pair reaction using a pDONR/222 vector and synthesis of cDNA followed by transformation into ElectroMAX™ DH10B™ through electroporation. Diluted (100-fold) library bacilli were cultured on LB solid medium (containing 100 mg·L^{-1} kanamycin) and the clones were counted after 12 h. Thirty-four clones were picked out for PCR detection with a universal primer pair for the pDONR/222 vector (F1/R1: GTAAAACGACGGCCAG/CAGGAAACAGCTATG AC).

A PureLink®96 Plasmid Purifification System (Invitrogen, Carlsbad, CA, USA) was selected to isolate mixed plasmids and then the pGADT7 vectors (prey plasmids) were used to perform the gateway reaction. As above, a confirmation of the yeast two-hybrid cDNA library was performed with a universal PCR primer pair for the pGADT7 vector F2/R2 (TAATACGACTCACTATAGGGCGAGCGCCGCCATG/GTGAACTTGCGGGGTTTTTCAGTATCTACG ATT). The isolation of library plasmids were subjected to yeast-two-hybrid screening.

Bait Plasmid Construction and Assays of Auto-activation

The cDNA sequences of AMPK (open-reading frame) were obtained by PCR and fused into the pGBKT7 vectors according to the manufacturer's instructions (In-Fusion Advantage PCR Cloning Kit). Sequencing was used to confirm the recombinant pGBKT7-AMPK plasmid.

To test the auto-activation of bait plasmid, pGBKT7-AMPK, and the negative control pGBKT7, were transformed into AH109 yeast cells separately. Transformants were incubated on SD/-Trp, SD/-Trp-His, SD/-Trp-His-Ade, and SD/-Trp-His-Ade+x-α-gal agar plates for 3-5 days at 30°C, and then diluted at different concentrations (OD_{600} = 0.6, diluted from 1 to 1:100). If the colonies containing pGBKT7-AMPK plasmid on SD/−Trp and SD/-Trp-X plates appears, it was confirmed that the bait had auto-activated. Then, different concentrations (0, 10, 20, and 30 mM) of 3-aminotriazole (3AT) added to the SD/-Trp-X plates were used to inhibit the auto-activation. Bait plasmids not showing auto-activation were utilized in screening by the yeast-two-hybrid system.

Supplementary Data

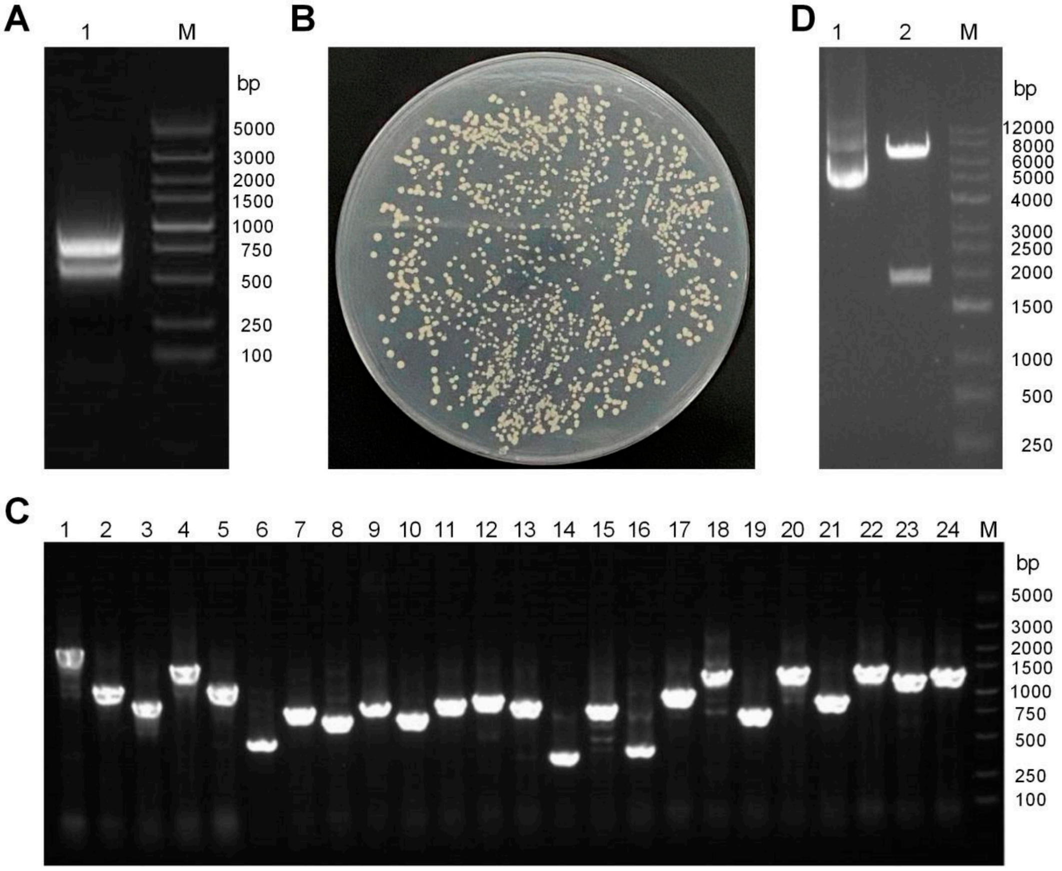

Figure S1. Quality tests of cDNA library and bait plasmid pGBKT7-AMPK. (**A**) Gel electrophoresis of mRNA of cDNA Library. (**B**) cDNA Library clones. (**C**) Gel electrophoresis of 24 selected clones of cDNA Library. (**D**) Construction of bait plasmid pGBKT7-AMPK. Lane 1: gel electrophoresis of bait plasmid; Lane 2: confirmation of pGBKT7-AMPK by digestion with *BamH I* and *Xho I*. M: DNA maker.

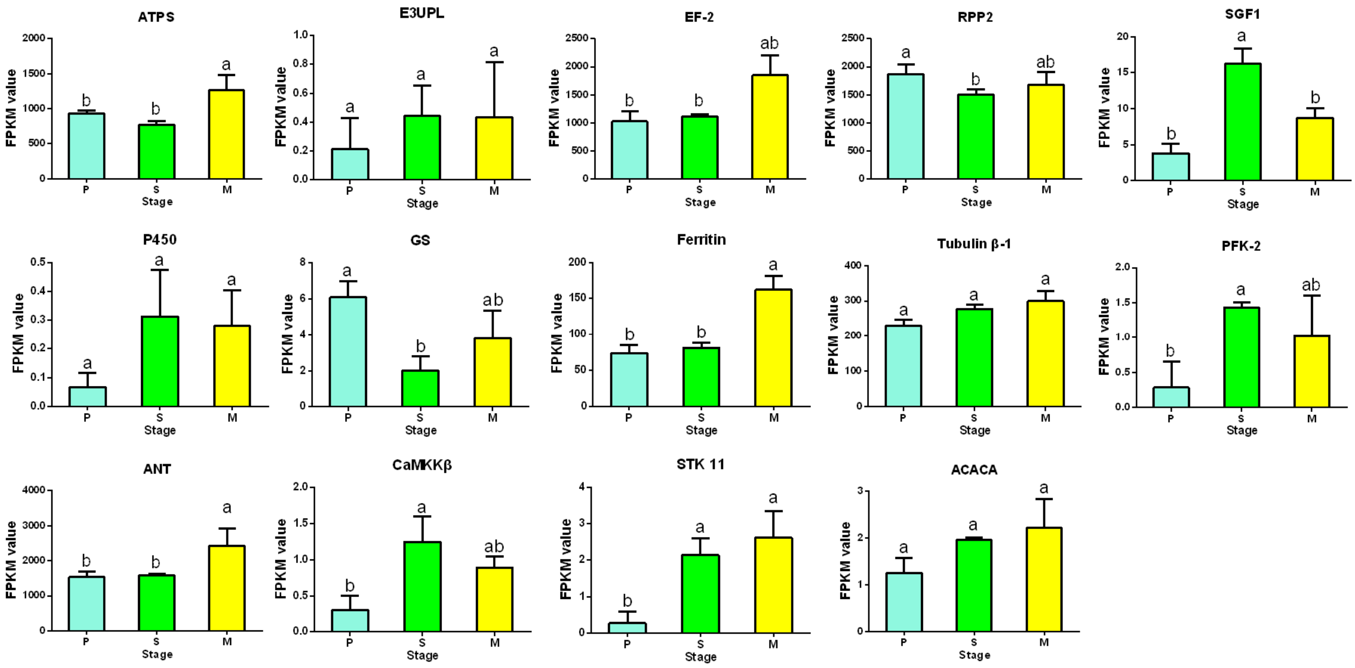

Figure S2. Transcriptomic analysis of mRNA expression levels of proteins interacting with AMPK during larval settlement. Different letters above the bars denote significant differences among treatments.

A

ATGGACCGGGAAGGCCGAAGGTTTGAGACCTCTATGGGCTCGGACATCCGACGTAGTCTGACCAACCTGACAAAGATGTTAAAAAATAAAATTCCTAGCACTGGTAAGAAAGTGAAGAGTGGAATGAAACCTTCGAAAGACCAGTTCACC
AGACAAGAGGAAATGGATCATCAAAAGCTGGCAGATTTTGTAAGGAATGAAAGTACCATGGTTAAACGAACCTGTAAAGCATATGTGCCGAGTAAGAAGGCCAAGGATGGAGCGTGTTGCTGTGGCCGTAGCAGGGAATGGCAAGGCCAT
TTGCCGTCCCCAGGCAGTGACGAACACATCTGGTCCCATAAAACTCACACACTGACGGAGAAAACTGACTCGTTTGGCGTTGTACAGTTTGAGGGATTTTGCAAGGAGACACTGAATGCACCTTACATCCGTCTGGGCCCGGACTCGG
ACCAGGAGCAGGTGTGGGACCTGCTGACAAAGCACTGGCACCTGCCCATTCCCAAACTGCTCATCTCCGTCACCGGCGGTGCGCAGAGGTTCGAACTCAACAACCGGCTCAAGCAGGTCTTCAAGCGCGGTCTCATTAATGCAGCT
GTCACCACAGGTGCCTGGATCATCACGGGCGGCACGGCAACAGGTGTGATGGAGTTTGTGGGTGAGGCGGTTCAGGACCAGATCATCACACGAATCAAGGGAGAGAACAAGGTGGTTGCCATCGGGGTTGCCACGTGGGGCTGCAT
CGCCAACAGGGAGGCACTGGACGGGCAGATGAACAACGAGACCAATGGCCTGTTCCCTGCCCACTACCTGGAAGATGACGTGGCCGAGATCCAGGGCAGGGACATCCCGCTAGACCACAACCACACTCACTTCCTATTGGTCGATG
ACGGCACCGAGGGCAAGTTTGGCGGCGAGATCGCATTCCGCTCGATGTTTGAGAGATACGTGTCGGAGAAAGTAGAGACTGGGGTGGCTGAAGCTCAGAGTGTGAACGTACCAGTGATCCAGCTGGTGGTGGAGGGTGGGGTAAACA
CAATGCTCACGGTGATGCAGGCGGTCGAGCAGAACATTCCCGTGCTGGTCCTGGACGGCTCCGGCAGGGCCGCAGACTTCATTGCTGAAGGATACAATCTCTCTAGGAACAGAAAAGATGAGGAGAAGTCCAGGTTCAGTAAAGAGTT
TGATGAGGACATGAAGGCGTATGCGGCCAGTATCTTCGCGTGGAAGGCCAGTGACGACAAGGAGAAGAAGATAGCAGAGTGCCTGCACCAGCTCAAAACAACCCTCGCCAAGCGAAAACTGGTGAATGTTTTCCGCATCAGTCAAGCA
GACACCAGGGATATTGACAGAGCCATTCTGTATGCATTGCTGAAGGCCAACAAGACGAATGCGAATGCCCAGCTGGCCCTGGCCCTAGCATGGAACAGGAGCGACATCGCCCGCCATGAGATCTTCACCCAGGCCAACAGGCACCAC
TGGAAGCGAAATCACAGAGGTCAGAACATTCCTCTGTACGATGCGATGTTCACGGCCCTGGTACAGGATCGCGTGGATTTTGTGCAGCTTTTCCTCGAAAATGGAGTCAGCCTGAGAAAGTTCCTGAGCATCGAAACACTCTGGAACCT
TTATGCCAACGTGCTGGATGATGAGTCAGACACAATGGCCCAGCTCCTTAACAACCTTATGTCCTACCTCAAGCAAACGTGGGGAGCGTACCTGTCGTGCAAGACGCAGAAGAGCAAGATTATTGACGGCGACCTGCTCGTCACCATC
GGCAAGCTAATGGTCCATCTCCTTGGAGACGAGAACTGCAACCCCTACCCCTCTGTCAAAGTCATTGAACCCAAACCAGAACCATATCCCCGATTTGCCCGGAAGGTGGCAACCTCAGCACCCCAGCCGACCCCCCGGCAGCAGTG
CAGTGTGTTCCAGTTGAACGTGGACAAGCGACCAACCATGGAATGGTTGCCAGGGAAACACGAGGAACAGATGACCACTTACAGCCGTCAGTCAAAGAAGGAATTTGGCGTTAAAGCTAAAGCTAAGCAAGCTGTGTCAATAAGTACGAT
ACAAGCAAAGAAGAAAGTACCCACCGACTTTGAGGACTACTGCTTTCGAGAGTTGTTCCTCTTTGCCATTCTGCTGAATCGACGAGAGCTCGGAAAACTGATGTGGCAGGCTGGTCAAGACCAGCTGGGCTCAGCCCTGATTGCGTGC
TCCCTGCTGAAAGCATTGTCCATCATAGCAGACGATGAAGAGGAGCTGGAGTTGTCCTTGGATCTCCTGGATCACGCAAATATGTATGAGGACATGGCCTACGGCGTGCTGTCCGAGTGTTACAAGCGAGACAAGCAGATGGCTCATC
AGCTGCTGGTGCGCAGACTCGAGTCGTTTGGCAACACGACGGTGTTCACGCTGGCCGACACCAACACGCTGATGAAGTTCATGGGTCACACCTGCTGCCAGACAAAGCTCAACCTCATCTGGAAGGGTCGCATGGCCCTCTACACAC
AGTCCTGGAAAATTTGCCTGTCGCTCTTGCTGCCATTTGTTATCCCGACCATCAAGTTCACGACCAGCGAGGGTGTGATGGAGAATGAGGAGGATGAGTATGATGACCTTGGTGAGGGGGAGGAGGAGGAGGTGGCAGCCAAAGAGGA
GGCACACGCAATATCTAACAAGGAAACACCAAATGACCCACATGATGGTAAAGATGACATTTTCATCCGATCAAAGACCCGAAGAAACAAGAAGCCTGCAAGAAAACTGTATAAGGTGCAGATGTTTGGAAATAGGAAAATGGGGACCATC
GGAATTCTTGACGCCTTCTACTACTTCTACACAGCACCTGTAACTGTGTTCACTGTCAACATGATATCATACCTGATCTTCCTGGGCCTGTTCTCGTACTTCGTGCTGGTGGACCTACAGGAAAAGACAACTGTGATAGAGTATGTCGTC
TGGGGCTGGACCCTCACCATGTTTGTGGAGGAAATCAGGCAGTTGATATCCAACGACAGACGCTCCCTCTGGTACAAAGTGCGGAGCTGGTACTCATCCCTGTGGAACAAGTTCGACCTCTCGATGTACCTCATCTTCATCCTGTCAG
TGATTCTGCGCTTCACCCTGTCTGGCGACAAGTTTGTTTATGCGCGCATATTCTACAGCATTACTGTGGCAATGTATTACCTGCGGTTTATGCAGACTTTCTTTGTGGAGAAGAACATAGGGCCAAAAGTCATCATGATCAAGAATATGTT
GATAGACCTGATGTTTTTTGTTGGGATATTCGTGGTGTTCCTGCTGAGCTTTGGCATCATGTACCAGGCGAATCTGTTCCCGAACTCTCCGCCCTCGTGGTACCTCCTAAAGAGCGTCGTCTACATGCCGTACTGGCAGATGTACGGC
GAACTCTTCTTGGAAAACCTTGAAGGAGACCCAACTGAGAGCTGCACCACCAACGAGAGCGTGTGGCGGGCAAACGGTGGTATTGACCGGTGCCCTGAAGACAATGCGCTGGTTCCCCTTCTAGGAGCACTCTACATGATCCTCACC
AACATTCTCCTGTTGAACCTTCTCATTGCCATGTTCAGCTACACATTCCAGATGGTACAGGACAAGTCCGAGCAGGTGTGGCGCTTCTACCGGTACAGCCTCGTGCACGAGTTCTACGAGCGGCCTGTGTTCTGTCCCCCTCTGATCA
CCCTGGCGCACATCTACCGCTGTGCCATATACCTGCTGAGGAAGGGCTGCAATAGGGGCCATTACAAGAACGAATTCAAGTTGAAGCTTGACGAGAAGGACAGCAACCGGGTGATCCTGTTTGTGCGCGGCGCCACAGAGAGCTACC
TCAACATCACCAAGCAGGCGGAGAAAGAGGAGCTCGGCAACAAAGTCACCGACACCGCCAACAGGCTTGAGAAGGTGATAGAGGAGTTAGACAACATCAAGGAGAGTGTGAAGGTGAACAAGATGTCGCCCATCCTGGACGAGAGCCG
CTCCACCATGGAGATGGACATGTCTGTGTCCTCTGCCAGCCCTAGGACCTTGAGCAGACGGACATCATCGGGTGTCAGCAGTGCTCTGGTGAGTCAGCTGCAGGACCAGGTGGACGATCTGAGTCAGCAGATGGCCGAGACAAGCG
CCCAGACGACACGGATCCTGGAGATGATGCAGATGCTCTGTCAACAGCACACGCTTGCACGCTCACACAAGGACCCCAGCTCATATATTTAA

B

ATGGGATCAGGAATAAGTGCCTTATGCAGTTCTGGAGAGTTTGGACCATGTTTCTCCAAGAAGAAGAGACCTCCGAAGATCGCCTCGCTCAGAAACACTCATTTCCAACAGAGCCAAGATCTTGAAGAGATGAGAACCAAGGGGCGGTT
GCTATCAGCAGGAGACCCACACGACACCACCTTCATACAGGAGCACAAGATCACGGCAGGCGGGAAGGCGCTGGAGGCAGAGACCAACAAGGAGAACACGTTTGAACACAAGCTTCTCAACCGGGATAGCGGCAATATCATATTGCTA
GAGGACGAGTGTATTCAGAAGGGTGATACTGACAATTCAAAAACAACTCTTGCAGGAAAACCAGTCAAATATCCATTGCATCGGAAGAGTTCGAACATCACAAATGACTCCGGACGGAGTACAGTTAGCGATCTTGGTGAAACTGAGAAT
GACTTGCAGACGGACCTAAATGAGAGTGAAAGCCATGTTTGTTGTCATAGTAACCATTTTGTTTCAATAGATCCACATCGAAGAGAGAGTTTAACTGTGATTGATTCCACTGGACACTTAACACTGCCATCGCCTATCGAATGCCTGTCAG
TGCGCCCGTTGCCGGGTGAGGACCTAGACTATCAGTTTGACAGCAGCGCCTGCTCGCCGTGTGAGCACGGTGCATGTGAGCGGCAGGTCTCCCCCACCCACGGGCGGCCAGCCAGCGGGGGACACCTCCACAGGGGGCATGCTC
CCAAAATACTCTACAGCAACTCAGAAGATAGCAAACTAACTCACCGTCGACTGTCTTACCCACGTTCACCTTACACATCGCCTACTGCGTCGCCACGGCTTCGTCGCCAGCCGACCATGGAGACAAGAAGAATCTCCGTGTCAGACAG
CTGTGATGGCTACATACAACTGAATCAATATAAGCTCAAGGATGAAATCGGCAAGGGTTCCTATGGTATTGTGAAGTTGGCCTACAATGAAGCAGATGATGCACATTATGCAATGAAAATACTTTCAAAGAAGCGATTGCGGAAAAAAGCA
GGCTTCTTTGGTCGGCCTCCGCCCTCCCGTGACGGCCGTTCCCCTGCGCGGCCCCCGACCCACCCCCTGGAGCGCGTGCACCGGGAGATTGCCATCCTGAAGAAGCTGGACCACCCCAACGTGGTGCGCCTGGTGGAGGTGCT
GGACGACCCCGAGGAGGACAACCTCTACATGGCATTTGAACTGGTGGAGAAAGGGGAGGTGATGGAGGTGCCCACCAAGCAGACACTGCCGGAGGAGAAGGCCTGGAGCTACTTCCGGGACATCGTCCTCGGCATAGAGTACTTAC
ATTATCAGAAGATCATACACAGGGACATCAAGCCATCAAATCTTCTGTTAGGAGATGATGACCATATCAAGATAGCAGATTTCGGCGTGAGCAACGAGTTCACAGGCAATGACATCTTCCTCACCAGCACAGCTGGCACTCCAGCGTTCA
TGGCTCCTGAGGCACTCAAGGAGGAGAAGGAGAACTTCAGTGGGCGAGCCCTGGACATCTGGGCCATGGGAGTCACCCTCTACTGCTTCCTCTATGGACGGTGTCCTTTTGAAGATGATATGCCACTGAGTCTGCACAAGAAGATCC
TCACAGACCCAGTGATATTCCCTGACAAGCCTGCTGTGTCAGAGGCTCTGAAAGATCTGATCCTTCAAATGCTCAACAAAGACCCAGACAAGAGGATTACATTGCCCAGCATCAAGGAGCACTGCTGGGTGACCAAGGAAGGCCAGTT
GCCATTGCCAACGGAGAGTGAGAACTGCATCCTGGTCACTGTGACGGATGAGGAGGTGGACAACGTGGTCAAACACATACCCAAGCTGGAGACCCTGATCCTGATCAAGAGTGTGCTGCGCCACAAGTCCTTCAAGCACCCATACAA
GGACCACATCAAGGAAGAGTTCCAGCGCTCTGGCCGCTCCAACTCCGCCCCTAACTCCACCTTTAACCAGGTGGTCAAACAGCGTCTGTCTGTGAGTGAGAACATTGAGGAGCAGTCCTAG

C

ATGGCTGACAAGAATCAACCGCAGGTCAAAATTGGCCACTATATTATCGGCGATACGCTCGGCGTTGGAACCTTTGGGAAAGTAAAAATTGCAACACATCAGCTGACTAACCATAAGGTAGCTGTGAAGATCCTAAATCGACAGAAAATC
AAGAGCCTTGATGTTGTCGGGAAAATAAGAAAAGAGATTCAGAACCTCAAATTGTTCAGACATCCACACATAATCAAGCTGTACCAAGTGATCAGTACTCCCACAGATATCTTCATGGTAATGGAATATGTGTCCGGCGGTGAACTGTTCG
ACTACATTGTCAAGAATGGAAAGTTAAAGGAGCAGGACGCGCGTCGGTTCTTCCAGCAGATTGTGTCCGGCGTGGACTACTGCCATCGCCACATGGTCGTCCACAGAGATCTGAAGCCAGAGAACTTGCTCCTCGATCACAACCTCAA
CGTGAAACTTGCTGACTTCGGGTTGTCTAACATGATGCATGATGGGGAGTTTCTGAGAACCAGCTGTGGCTCACCAAACTATGCAGCCCCGGAGGTGATTTCTGGAAAATTGTACGCCGGACCTGAGGTGGATGTATGGAGCTGTGGT
GTCATCCTGTATGCTCTGCTGTGTGGAACGCTGCCATTTGATGATGAACATGTTCCCACATTGTTTAGAAAGATTAAATCGGGCATCTTCAACATCCCCGAGTACCTGAACAAGGAGGTGGTATCACTGCTCTGTCACATGCTGCAGGT
GGACCCGCTCAAACGGGCCACCATGAAGGACATCAGGGAGCATGACTGGTTTAAGAAGGACCTGCCAGCTTACCTGTTTCCCCCGCCCAGTGACCACGATGCCTCCATCGTTGACATCGATGTTGTCAGGGAAGTGTGCGACAAATT
CGGGGTGCAGGAATGGGAGGTTGAGGCGGCCATCTTGGACGGAGATCCCATGGACCAGCTCGTGATAGCGTACAATTTGATCCTCGATAACAAGCGCATTGTAGATACAGCGAGCTCTGTTAGTTTCCAAGATTTCTACTTGGCTTCC
AGCCCACCTAGTGATGCTTTCTTGTTGGAACAGACTGTAAAGCTGAGCTTGACAAAGGGAGCAGTCGTAAAGCGCCAGCTGTCGACAAGTGGGTTAAAGCCGCATCCAGAAAGAATGCCAGAAATGAAAGACGTCACCCAGACGTTGG
ACCCTTTGCTGACAACAGTAAATAAAGCACAGACCCCAGCCAAGAAAGCCAAATGGCATTTAGGTATTCGATCCCAGAGCAAGCCGCAGGACATCATGCACGAGGTGTTCCGAGCGATGAAAACATTGGGCTTTGAATGGAAGGTTGTG
AATAACTACCACGTCCGAGTGCGGAAATTGAACCCCGTCACCAACAAACACACCAAGATGAGTCTCCAGCTGTACCAGGTGGACACAAAGAGTTTCCTGCTTGACTTCAAAAGCCTTCTCGATAACGAGGAGAACGGTTCCCTGCCTA
CCGTAGCCAGCATGGCCTCCCGGGACAGCTCCGACAATGTTCTCTTCGGAAATCGTAGTTTCTCAGTATTTGAAAAAGAGGACAAAATGGACGTCGATGAGCCACCAAAGAGCCACCACACGCTGGAGTTCTTCGAGATGTGTGCCCA
GCTGATTACCACCCTTGCCAGGTAG

D

ATGTACGAGAAGCAGACGGCGTCGGAGTACCCGCCGGCGGGGGACTACCTGACCATGCTGTCCAAGTCCACGTACGACCCCGTCAAAACAGCGTACGACTCGAGCGGCTACCCGATGTCCTCCATGGCGAGTATGAACTCCATGT
CGATGGGTTCCATGGGCGGAATGGGCACCATGGGCTCCATAGGGTCCATGGGTATGAACAACATGAACTACTCCACACCGGCGCTCACCGGGATGGGCGCGCCGCACGGCTACGGCATGGGTCCCGGTGTCGGGATGACCGGCG
GAATGTACGGATCCATGAACACAATGAACACCAACGGTCACATGAGCGGGATGACCAGCCCAGTTCCCGGCATGAACGGCATGTCAGAAGCCATGTCTCCTATGCGAATGGATATAAACCGGGCTCAGGCCATCAACCGAGCGCGG
GAGAAGACCTACCGGCGGAGCTACACCCACGCAAAGCCGCCGTATTCGTACATCTCACTCATAACTATGGCAATACAGCAATCTCCGAACAAAATGTGCACATTAAGTGAGGTGTACCAGTTTATTATGGACTTGTTTCCGTTTTACCG
ACAGAATCAGCAACGCTGGCAGAACTCTATCCGCCATAGTTTATCATTCAATGACTGTTTCGTGAAAGTGCCGCGAACGCCGGACAGGCCCGGGAAGGGCAGCTACTGGACTTTACACCCGGACTCCGGGAACATGTTCGAAAATGG
TTGCTACTTGCGGCGACAAAAGCGCTTCAAATGCATCAAGAAAGAGAAGATCCGAAAGTCACCTGGCGAGGGAGGTATGGGATCAGACGACGAAGATCAGGATGAATCCGGCTGTCAGTCTGATTGCGATTCAGGGCCAGGGTCACC
ACCGGTGGAGTCGCAACCAAATCATCATCAGCCACCGCCCCAGCATCAGCAGCACCAGCAGCAATCTCAGCAGCAGCAGCCGCAGCCACCTCATCACATGTCCAACCAGCTGGAGCCCCTACCACCGAAGTCGGAACCAGCGTCG
GTCGGCACACCAACTTCCGGTTCGACGCCGGGACCGGATATGCGGCATCTACACCAGCACCATGACGTCATGTCGATGACCCACGGCTACCCGGGTGTGAACCCGACCCACTCATTCAATCATCCGTTCTCAATAAACAATTTAAT
CACGGAACACTCAAAAATGGACTTGAAACTCTATGAAATGCAGGGCATGTACCCGGCGTATAATAATATGCACCAAATGAACATTCCACCAAAAGTTGAAGGGTTACCCCCGATGACTGCGGACGGTGGATATTACAAGACTTATACCC
CTCACAGCACAGCCAGTCTGTGA

Figure S3. Open reading frame sequences of *TRPM7* (**A**)**,** ***CaMKKβ*** (**B**)**,** ***AMPK*** (**C**)**, and** ***SGF1*** (**D**) from *M. sallei*.

Table S1. List of sequences of primers used for qRT-PCR.

Gene name	Forward primer (5′—3′)	Reverse primer (5′—3′)	Product size (bp)
β-actin	GTCACGGACGATTTCACGC	CCATCTACGAAGGTTACGCTCT	143
TRPM7	AGAGTATGTCGTCTGGGGCT	CTTGTTCCACAGGGATGAGT	124
CaMKKβ	GCTTCCTCTATGGACGGTGT	TTGATGCTGGGCAATGTAAT	178
AMPK	AGGAATGGGAGGTTGAGGC	GGGTCTGTGCTTTATTTACTGTTG	324
SGF1	GCGAGGGAGGTATGGGAT	GACTTCGGTGGTAGGGGC	214

Table S2. Sequences of siRNAs.

Name	Sense（5'—3'）	Label
TRPM7-1	GCUCGAUGUUUGAGAGAUA	5′ Cy5
TRPM7-2	CCAGCUCCUUAACAACCUU	5′ Cy5
SGF1-1	CCAUGUCGAUGGGUUCCAU	5′ Cy5
NC-1	CCAGCUGUAUGGCUUGCAU	5′ Cy5
SGF1-2	CCAUGUCUCCUAUGCGAAU	5′ Cy5
NC-2	CCACUCUUAUCGCGGUAAU	5′ Cy5
